# The Third Month’s Development Predicts the Side and Oblique Sit and Walking

**DOI:** 10.3390/jcm14238492

**Published:** 2025-11-30

**Authors:** Ewa Gajewska, Joanna Surowińska, Michał Michalak, Jędrzej Gajewski, Anna Chałupka, Mariusz Naczk, Alicja Naczk, Magdalena Sobieska

**Affiliations:** 1 Department of Developmental Neurology, Poznan University of Medical Sciences, 60-806 Poznan, Poland; joanna021@icloud.com (J.S.); jedrzejgajewski99@gmail.com (J.G.); 2 Department of Computer Science and Statistics, Poznan University of Medical Sciences, 60-806 Poznan, Poland; michal@ump.edu.pl; 3 Collegium Medicum, University of Zielona Gora, 65-417 Zielona Góra, Poland; 4 Department of Rehabilitation and Physiotherapy, Poznan University of Medical Sciences, 60-806 Poznan, Poland; achalupka@ump.edu.pl (A.C.); msobieska@ump.edu.pl (M.S.); 5 Institute of Health Sciences, Collegium Medicum, University of Zielona Gora, 65-417 Zielona Góra, Poland; m.naczk@cm.uz.zgora.pl; 6 Department of Physical Education and Sport, Faculty of Physical Culture in Gorzow, 61-871 Gorzów Wielkopolski, Poland; a.naczk@awf-gorzow.edu.pl

**Keywords:** motor performance, infant, quantitative assessment, qualitative assessment

## Abstract

**Background/Objectives:** Motor development is endogenously generated until approximately the third month and strongly approximated in all children; hence, this is an excellent time to assess development, both in terms of the achievement of selected milestones (quantitative assessment) and individual single motor elements (qualitative assessment). This article aims to identify which selected milestones and single motor elements performed at the third month and between the fourth and fifth months condition the achievement of the side sit and oblique sit, followed by walking. **Methods:** All the children (93 infants) underwent prospective evaluations of motor development at 3, 4–5, and 7–8 months of age, with final assessment at 12 months. The three-month development analysis was based on the Quantitative and Qualitative Assessment of Motor Development Worksheet. **Results:** All the quantitative features showed a statistically significant effect on achieving side and oblique sitting and eventually walking at 12 months, with those studied between the fourth and fifth months being stronger predictors. The distal parts of the upper limbs analyzed in both positions at three months of age have a strong relationship with the side sit, the oblique sit, and walking. **Conclusions:** Quantitative and qualitative assessments in the third month have predictive value for achievement of side sitting, oblique sitting, and walking. Correct distal upper limb function development is necessary to achieve side and oblique sitting.

## 1. Introduction

Recent research indicates that the third month after birth (i.e., after two months and before the fourth month of life) is the moment when we observe a significant change in motor development, from development determined mainly genetically to development influenced by internal and external factors, also (e.g., environmental) [1,2,3,4]. Thus, it can be assumed that motor development is endogenously generated until approximately the third month and strongly approximated in all children. Hence, this is an excellent time to assess development, both in terms of the achievement of selected milestones (quantitative assessment) and individual partial motor elements (qualitative assessment) [3,4,5]. Analysis of motor development at the third month of an infant’s life has also become essential for other reasons. Early initial diagnosis of cerebral palsy is generally made before five months of age. Using General Movements Assessment (GMA), Hammersmith Infant Neurological Examination (HINE), and Magnetic Resonance Imaging (MRI), infants with detectable neonatal Cerebral Palsy (CP) risk factors can now be diagnosed as early as three months of age (corrected for prematurity), with a sensitivity as high as 97% [6,7,8,9]. The diagnostic approach developed by Vojta involves both a milestone assessment and a qualitative assessment. According to this approach, each milestone is carefully analyzed in terms of the quality of function performance. According to the Vojta concept, correct motor development at three months is summarized as “quadrangle of support” in the supine position (support occurs at the head, shoulder blades, and pelvis) and symmetrical elbow support in the prone position (“triangle of support”—elbows and pubic symphysis). A stable symmetrical position with spinal uprightness and good head control is associated with accomplished cranio-caudal development at three months of age [4,10]. By the third month, the child should have mastered a stable position of symmetrical support, both in the supine position and the prone position, and further development requires mastery of additional asymmetrical positions and forms of movement. The first such skill involves turning to the side and providing asymmetric support on the elbow, which is usually achieved between 4 and 5 months of age. This foreshadows an increasingly proficient move toward locomotion, manual skill refinement, and spinal rotation, which is required for alternate crawling movements or walking [4]. Assessment of turning to the side and asymmetric support on the elbow is also often analyzed at developmental scales as an essential moment of change. In the Alberta Infant Motor Scale (AIMS), asymmetric support is described between 5 and 7 months of age as reaching from forearm support [4,11,12]. The importance of these listed functions is indicated by the fact that they are also analyzed via the scale Gross Motor Function Measure (GMFM) in children—for example, for diagnosis of CP [13,14]—as one of the primary functions in the sample analysis: lying and turning.

The next stage of improvement in motor skills is the gradual detachment of larger and larger body parts from the ground, thus effectively overcoming gravity, moving on to locomotion, and becoming increasingly proficient in manual activities. A stable position on the side (the side sit) appears in motor development in body rotation from a supine position to a prone position and results from the need for a purposeful upward grasp. The base of support of a stable position on the side is formed by the forearm, thigh, and pelvis and is shaped like a trapezium. As a result of this course of movement, the child moves to a stable position on the side at approximately seven months of age, followed by an oblique sit, which leads to the achievement of crawling. The commonly used AIMS also includes a side sit described as “propped lying on the side”, where the child weighs on the elbow, forearm, leg, and one side of the trunk which, as Vojta indicated, occurs from 7 to 9 months of age. The oblique sit, defined as four-point kneeling to sitting and half-sitting, occurs between 7.5 and 10 months of age [4,11,12]. The final step in the analysis of motor development, in terms of its milestones, is often the achievement of independent walking by the child.

## 2. Materials and Methods

This study aims to identify which particular motor elements assessed at the third month, as well as between 4 and 5 months, condition the performance of the side sit and oblique sit, followed by walking.

### 2.1. Participants

The tests were performed at the Clinic of Neurology between 2018 and 2023, where parents with children referred by a general practitioner or pediatrician came to assess motor development, in some cases due to concerns of the parents themselves. Some parents appeared for a screening even if they did not have any concerns. The motor development of children born prematurely was analyzed at the corrected age [15]. The included children were assessed at all depicted time points. Those lost for follow-up were not included.

Inclusion criteria:

The study group consisted of children born at term and prematurely (between the 28th and 42nd weeks of gestation). All children presented with their parents to the Neurology Clinic for an assessment of motor development. These were children referred by a pediatrician, family doctor, or due to parental concerns, such as developmental asymmetry or poor head control.

To determine the study sample size, data on the number of births in a given area were previously collected; the required sample size was 383. The Statistica.pl software (Statistica 12.1) was used, assuming the population of the region where the study was performed and the expected percentage of children affected with motor disturbances. Considering the number of children with motor development disorders, 100 children were included in the group. The final number of children was 93 because not all returned for follow-up examinations.

Exclusion criteria: The exclusion criteria included extreme preterm birth (below the 28th week of gestation), genetic or metabolic disorders, severe congenital disabilities, and children with microcephaly or macrocephaly.

All the children underwent prospective evaluations of motor development at 3, 4–5, and 7–8 months of age. The final assessment of motor development was performed at 12 months of age. The development analysis at three months of age was based on the Quantitative and Qualitative Assessment of Motor Development Worksheet, which has already been described in previous studies [5,16].

#### Ethics Statement

The study was conducted at the Center for Child and Adolescent Neurology Clinic between 2018 and 2023, following the ethical guidelines of the 1964 Helsinki Declaration and its later amendments. The children recruited for the study were patients/clients of the Child Neurology Center. All parents/caregivers agreed to participate in the study, as, apart from routine assessment and therapy, no extra visits were necessary. Parents/caregivers of the children studied gave written consent to participate in the study. The Research Ethics Committee at the Poznan University of Medical Sciences, Poland, approved the study and registered it under number 22/10 (7 January 2010).

### 2.2. Procedure

An experienced physiotherapist, blinded to the clinical data, assessed all 93 infants at three months (at least 12 weeks completed; in the case of preterm babies, corrected age was considered). Each infant was warm, fed, not sleepy, not tired, placed on a large rehabilitation table, always in the same premises, and the assessment was made in the morning hours. The assessment of motor performance was performed in the prone and supine positions according to the “Quantitative and qualitative assessment sheet”, used in previous studies [5,16].

Each element was assessed as zero if an infant performed it only partially or entirely incorrectly, or one if an infant performed it entirely correctly. The duration of the examination was between 10 and 15 min. Each element had to be observed at least three to four times during the assessment.

#### 2.2.1. Quantitative Assessment in the Third Month of Life

This assessment consisted of a quadruple of support in the supine position (head in the axis of the body, upper limbs aimed at the centerline, lower limbs flexed up to 90 degrees in the hip and knee joints, foot in an intermediate position), and symmetrical support on elbows in the prone position.

#### 2.2.2. The Qualitative Assessment at the Third Month of Life

The physiotherapist’s assessment was based on a self-designed motor performance sheet (quantitative and qualitative assessment sheet) at 3 months of age. The children were observed in the supine and prone positions. A qualitative assessment included 15 elements in the supine position and 15 in the prone position. All elements were described in the previous paper [5]. An infant could score a maximum of 15 points in both the prone and supine positions. Both sides were assessed for symmetrical parts of the body to exclude asymmetry. The quantitative and qualitative assessment sheet was previously tested for reliability; the interobserver reliability ranged from 0.870 to 1.000, whereas the intraobserver reliability was equal to 1 [16]. Both reliability tests were performed on recordings taken during the examination. A comparison between physiotherapeutic and neurological assessments showed high agreement, with high conformity coefficients (z = −5.72483, *p* < 0.001) [16]. The examinations were performed independently. Both the neurologist and the physical therapist knew only whether the child was born prematurely or at term, but were not aware of the infant’s medical history details or the parallel opinion.

The neurological development assessment was carried out according to the comprehensive neurological examination. Neurological examination was based on the Denver Development Screening Test II (DDST II), along with the evaluation of reflexes, muscle tone (hypotonia and hypertonia), and symmetry. DDST II covers all areas; however, this research involved two evaluation parameters: small motor skills/precision and adaptability, and movement and posture coordination/large motor skills.

Following the examination, the neurologists classified the infants into one of three groups: “normal” (no neurological abnormalities), “suspect”, and “abnormal”. The child was classified as “abnormal” when showing distinct neurological disorders, such as increased (hypertonia) or decreased (hypotonia) muscle tone in combination with abnormal reflexes, and if they failed to complete the motor tasks for their age group in the DDST II. The children were classified as “suspect” when they exhibited mild neurological disorders such as mild problems with muscle tone control, slight reflex abnormalities, minor developmental asymmetry, and delayed motor development in the DDTS II.

Two physiotherapists carried out the interobserver examination using quantitative and qualitative assessment sheets independently on the same day, and the results were kept blinded until final statistical analysis. Forty children were assessed via inter-observer examination. The intraobserver portion was performed by comparing direct observations with the outcome of video recording analysis involving 44 infants performed at two-week intervals. The observer did not know the clinical status of the infants. Inter-observer and intra-observer reliability were examined and showed strong reliability (kappa = 0.876 and 0.871, resp.) [16].

Quantitative assessment at the age of 4–5 months

All children were subsequently assessed at 4–5 months; turning to the side was checked in the supine position, and asymmetrical support on one elbow was checked in the prone position.

An infant could score 1 for each feature, both in the prone and in the supine position.

#### 2.2.3. Quantitative Assessment at the Age of 7–8 Months

Between 7 and 8 months of age, the physiotherapist assessed if a child performed the side sit and the oblique sit. An infant could score 1 for each feature.

#### 2.2.4. Quantitative Assessment at the Age of 12 Months

At 12 months, the physiotherapist assessed if a child walked independently (moved quickly with short steps), and a delay or suspicion of having CP was noted (a neurologist confirmed the diagnosis at 18 months).

## 3. Statistical Analysis

The mean with standard deviation was used to describe interval variables after a preliminary assessment of the normality of the distribution with the Shapiro–Wilk test. A logistic regression model assessed the relationships between assessment items and milestone achievement (side sit, oblique sit, and walking). The model included sex and prematurity as control variables for potential confounding effects. Model fit statistics (likelihood ratio chi-square test, *p*-value, pseudo R^2^) were reported to assess goodness of fit. Results were presented as odds ratios (ORs) with corresponding 95% confidence intervals (95%CIs) and *p*-values. All tests were considered statistically significant at *p* < 0.05. All tests were performed using Statistica.pl, version 12.1.

## 4. Results

The analysis was performed on 93 children (50 boys and 43 girls). Of these, 69 children were born on time, while 24 were born prematurely [17]. Most subjects were born in good condition, with Apgar scores ranging from 8 to 10 points. The demographics are shown in detail in Table 1.

All the studied children performed the side sit and the oblique sit to one side or the other, fully symmetrically. Alternatively, they did not do it to either side. Therefore, the diagrams and tables included only the performance of this element and not the side. Eighty-three out of 93 children performed both the sit and the oblique sit, or neither; eight children did not perform the oblique sit, although they did the lateral side sit, while two performed only the oblique sit.

**Figure 1:**
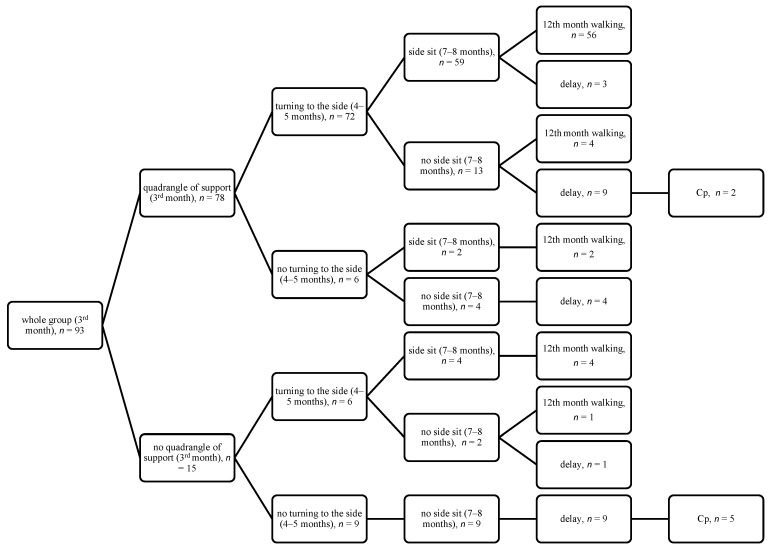
Quantitative features in the supine position at 3 months, 4–5 months, and 7–8 months (the side sit) with final assessment at 12 months.

**Figure 2:**
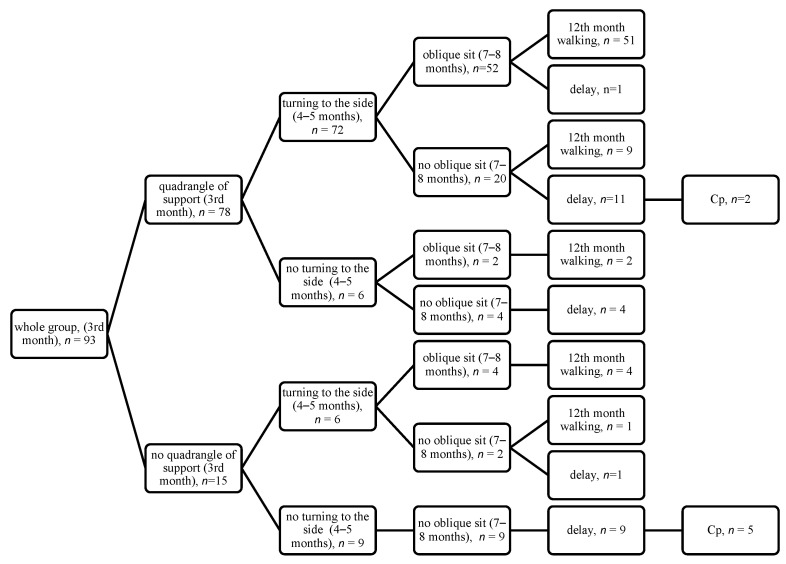
Quantitative features in the supine position at 3 months, 4–5 months, and 7–8 months (the oblique sit) with final assessment at 12 months.

**Figure 3:**
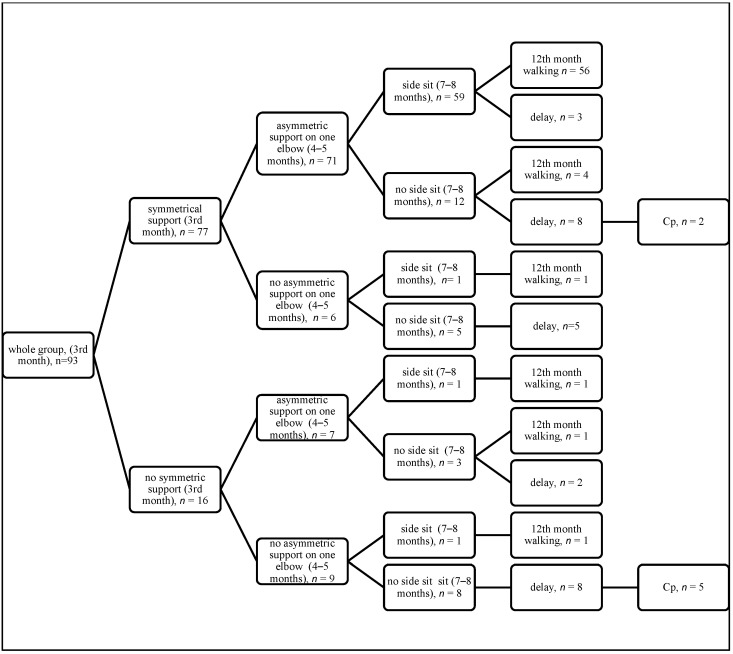
Quantitative features in the prone position at 3 months, 4–5 months, and 7–8 months (the side sit) with final assessment at 12 months.

**Figure 4:**
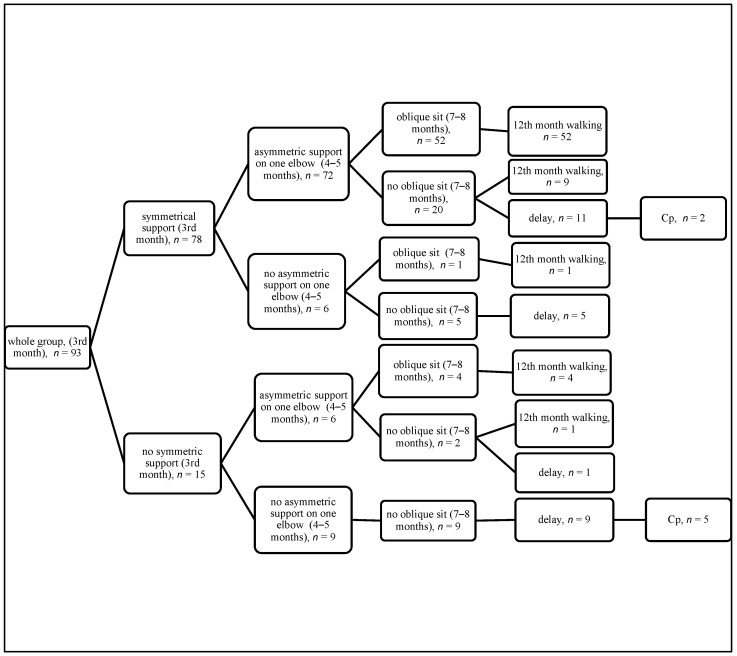
Quantitative features in the prone position at 3 months, 4–5 months, and 7–8 months (the oblique sit) with final assessment at 12 months.

The results of quantitative assessment (motor features) are summarized on the diagrams, showing the achievement of appropriate features from third month (the quadrangle of support in the supine position, the symmetrical support on both elbows in the prone position), then 4–5 months (turning to the side in the supine position, the asymmetric support on one elbow in the prone position), then 7–8 months (the side sit and the oblique sit), with final assessment at 12 months (walking) (Figure 1, Figure 2, Figure 3 and Figure 4).

As the statistical values show, none of the analyzed regression models achieved statistical significance for prematurity or sex. On the contrary, almost all regression models for quantitative and qualitative assessment variables showed high significance. The most significant results (with the highest OR values) are marked in gray in Table 2, Table 3 and Table 4.

The strengths of the impact (statistical effect) for the quantitative features from the 3rd and between the 4th and 5th months on achieving sits and then walking are shown in Table 2. All the quantitative features analyzed showed a statistically significant effect on achieving the sits and eventually walking at 12 months of age. The quadrangle of support in the third month strongly impacted the side sit. The features studied between 4 and 5 months were stronger predictors for achieving the side sit, the oblique sit, and walking than the elements studied at the third month.

If a child achieves correct development at three months and between months 4 and 5, then they have a 71.8% chance to achieve walking at 12 months. If they do not develop correctly at three months but make up for their development between 4 and 5 months, then the chance of walking is still high (4/6 children, see Figure 1, Figure 2, Figure 3 and Figure 4). This observation is confirmed by the high odds ratio for asymmetric support on the elbow and turning to the side occurring at 4–5 months; however, abnormal development at three months is a strong warning of developmental delay at 12 months. Among the 15 children who failed to demonstrate this ability, as many as 5 (30%) were later diagnosed with cerebral palsy (CP), as shown in Figure 1, Figure 2, Figure 3 and Figure 4.

We also noted two children who developed typically until 4–5 months, but no longer sat by 7–8 months. Both showed a delay in the 12th month. At the age of 18 months, the neurologists diagnosed one child with hemiplegia and the other with diplegia. The reasons for late-onset CP remain unclear. Early motor delays may indicate CP risk, but diagnosis must involve multidisciplinary assessment.

The effects of qualitative features from the third month, in the supine and prone positions, on achieving sits and walking are shown in Table 3 and Table 4, respectively. Out of the elements assessed in the supine position, the position of shoulders and wrists and the position of pelvis and lower limbs had the most substantial impact on the sits and walking. In contrast, the symmetry of the head impacted the sits only. In the prone position, the open palms (and thus the symmetrical support on both elbows) were a powerful predictor of both sitting and walking. The position of the pelvis also contributed markedly to the achievement of all investigated elements.

It may be concluded that the stable position of the whole trunk, with good symmetrical support, is the basis of further asymmetric motor features (achieved between 4 and 5 months), and they contribute to more complex motor achievements such as the side sit, the oblique sit, and finally walking.

## 5. Discussion

The assessment of motor development should not be limited to examining so-called milestones; i.e., assessing whether the child has achieved specific complex motor activities on time. However, it should examine simple elements that make up these complex activities. According to Vojta’s concept, such an assessment system, called qualitative assessment, allows for detecting minor irregularities and, consequently, for better therapy planning and individually selected exercises. Our work shows that assessing such minor elements allows for predicting the achievement of complex motor activities, often many months later. At the same time, Vaclaw Vojta and other authors pointed out a long time ago what is increasingly discussed in the literature on the subject: that spontaneous motor skills (motor performance) in the third month of life [3,4] predict the correct or disturbed motor development. The consequence of this approach was the creation of the quantitative and qualitative assessment sheet, which assesses individual motor elements in the supinated and pronated positions and allows for the prediction of further motor development with high reliability [5].

Children’s activities, such as postural, locomotor, and manual activities; exploratory activities; and social interactions, involve motor skills [18]. For many years, it was thought that all motor development was innate. However, it is now believed that motor development is influenced not only by the characteristics of the child himself or herself, such as body weight and muscle strength, but also by environmental factors (housing conditions, presence of toys, family) [3].

By and large, it consists of the transition from endogenously generated varied movements that primarily serve exploration and sculpting of the nervous system to movements that can be increasingly varied and adapted to the constraints of the environment. This progress is manifested, for example, by the fact that for the first three months, infants learn to stabilize the head on the trunk [19]. At three months, there is a desire to grasp objects [10,20]. Infants direct about one-third or half of their hand movements to the face. They do this spontaneously, and when an object is put into their hand [10,21].

There are also reports showing that in the case of children with autism spectrum disorder (ASD), the first failure to master milestones in the first three months of life may be significantly associated with more than double the risk of a later diagnosis of ASD [22] but also the potential for early diagnosis of cerebral palsy [8,23].

Some previous studies have shown the validity of analyzing motor development at three months of age and its impact on achieving the functions of asymmetrical support on one elbow, rolling, sitting, crawl position, crawling, standing, or walking [16].

In addition, in an earlier analysis, we focused not only on the achievement of milestones at three months of age but also on the quality of motor development via the validated Quantitative and Qualitative Assessment of Motor Development Worksheet [5,16]. We also used it to analyze the prediction of achieving side sitting, oblique sitting, and walking, showing high predictive value. This information is essential for doctors, physiotherapists, or parents to introduce early physiotherapeutic interventions and reduce or eliminate delays. However, analyzing qualitative characteristics from a therapeutic point of view seems more important, as more precise therapeutic intervention is possible. Both the side and oblique sits are strongly related to the work of the upper extremities. Both of these positions of the child appear in rotation from the supine to the prone position [4] and arise from the need to deliberately grasp, for example, a toy in the air. A stable lateral position is maintained in motor development in the supporting limbs, leading to the development of the obliqued position, enabling the transition to crawling [4].

Our study revealed that for the development of these functions (the side sit and the oblique sit), a proper head positioning, a stable support of the whole trunk (both in supine and prone positions), the proper position of pelvis and the correct position of upper limbs is necessary at three months of age. Notably, abnormal development as early as three months of age affects a high percentage of children who will develop cerebral palsy. However, milder forms of cerebral palsy, which may reveal abnormalities later, cannot be excluded.

We realize this assessment is subjective (like other motor development scales), but it can be used in clinics. This assessment system is widely used among physiotherapists and doctors. Previous work emphasized that the correct attainment of all milestones is first conditioned by the alignment of the spine, shoulder girdle, and pelvis (thus, the proximal elements). Only when these are correctly achieved is the influence of the distal elements revealed; in other words, delayed quadrangle of support or symmetrical support, but then achieving asymmetrical support on one elbow, guarantees the achievement of subsequent milestones, including walking. While grasping and supporting functions seem to depend more on the proper functioning of the distal parts of the upper limbs at the third month, turning to the side between 4 and 5 months of age—which is likely related to the mastery of spinal rotational functions and the later function of alternating lower limb movement—appeared to be the strongest predictor for walking.

## 6. Conclusions

Quantitative and qualitative assessment in the third month has predictive value for achieving side sitting, oblique sitting, and walking. Asymmetrical support on one elbow and turning to the side guarantees the achievement of the side sit, the oblique sit, and walking on time. Proper head positioning, symmetrical support, proper position of pelvis, and correct upper limb function development at an early stage are necessary to achieve the side and oblique sitting.

We recommend performing quantitative and qualitative motor development analysis at 3 months as screening tests. Early detection of disturbances should evoke careful monitoring and/or appropriate physiotherapy.

### Study Limitations

This study has several limitations. First, the relatively small sample size (N = 93) reduces the precision of the logistic regression estimates, leading to wide confidence intervals for some odds ratios. This limitation is further compounded by the uneven distribution of cases across predictor categories, which can increase variability and limit statistical power. While significant associations were identified, the wide confidence intervals indicate uncertainty about the exact magnitude of effects and highlight the need for cautious interpretation. Future research with larger and more balanced samples must confirm these findings and provide more precise effect estimates.

The presented assessment system is still little known in the literature, but very widely used by physiotherapists. The assessment requires experience in working with infants.

Extremely preterm infants were excluded from the study due to the small size of the study group during the observation period. The research was carried out in only one clinical center.

## Figures and Tables

**Table 1:** Demographic data.

Parameter	Whole group, *n* = 93
Sex	boys, *n* = 50; girls, *n* = 43
Born	at term, week 39 ± 1, *n* = 69; preterm, week 33 ± 3, *n* = 24
Body mass	born at term 3470 ± 428/ preterm 2044 ± 730
Delivery	Vaginally, *n* = 64; Cesarean section, *n* = 24; forceps, *n* = 4, vacuum, *n* = 2
Apgar score at 5th minute	Good condition (8–10), *n* = 88; semi-severe condition (4–7), *n* = 4; severe condition (0–3), *n* = 1
IVH	I°, *n* = 2; II°, *n* = 3; III°, *n* = 3; IV°, *n* = 1
RDS	*n* = 9
hypotrophy	*n* = 2
hyperbilirubinemia	*n* = 2

Legend: IVH—intraventricular hemorrhage, RDS—respiratory distress syndrome.

**Table 2:** The influence of the main motor skills (quantitative assessment) in the 3rd month of life, in the supine and prone position, on achieving of the side sit and the oblique sit in the 7–8 months of life and final assessment at 12 months (walking). The results were adjusted for prematurity and sex; *n* = 93. The most significant results (with the highest OR values) are marked in gray.

Elements of Quantitative Assessment	Side Sit OR and 95%CI; *p* > [z] 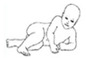	LR chi2 (3) Prob > chi2 Pseudo R2	Oblique Sit OR and 95%CI 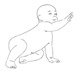	LR chi2 (3) Prob > chi2 Pseudo R2	Final Assessment at 12 Months—Walking OR and 95%CI	LR chi2 (3) Prob > chi2 Pseudo R2
Supine position 3rd month quadrangle of a support Prematurity Sex 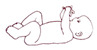	20.2 (3.8–107.8); <0.001 0.3 (3.8–107.8); 0.102 1.0 (0.3–2.6); 0.923	13.43 0.0038 0.1180	9.1 (2.2–37.4); 0.002 0.5 (0.1–1.7); 0.281 1.0 (0.4–2.6); 0.921	11.47 0.0094 0.0939	31.8 (3.8–269.7); 0.001 0.9 (0.1–0.8); 0.032 0.8 (0.3–2.3); 0.711	19.74 0.0002 0.1791
Supine position 4–5 months—turning to the side Prematurity Sex 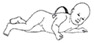	29.2 (5.8–147.8); <0.001 0.7 (0.2–2.6); 0.582 1.2 (0.4–3.4); 0.799	26.09 <0.0001 0.2293	18.2 (3.7–88.4); <0.001 0.9 (0.3–2.8); 0.715 1.2 (0.4–1.2); 0.860	19.67 0.0002 0.16.11	46.5 (7.7–282.1); <0.001 0.3 (0.1–1.4); 0.127 1.0 (0.3–3.2); 0.972	31.13 <0.0001 0.2824
Prone position 3rd month—symmetrical support on elbows Prematurity Sex 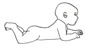	10.2 (2.7–38.1); 0.001 0.5 (0.1–1.7); 0.248 0.9 (0.3–2.5); 0.891	14.09 0.0028 0.1238	2.8 (0.9–8.9); 0.075 0.8 (0.3–2.4); 0.740 1.0 (1.4–2.5); 0.927	3.26 0.3537 0.0267	11.0 (2.7–45.1); 0.001 0.2 (0.1–1.1); 0.061 0.8 (0.3–2.3); 0.709	14.63 0.0022 0.1327
Prone position 4–5 months—asymmetric support on one elbow Prematurity Sex 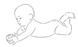	27.9 (5.6–138.2); <0.001 0.9 (0.3–3.2); 0.904 1.2 (0.4–3.5); 0.736	25.79 <0.0001 0.2266	18.0 (3.7–87.3); <0.001 1.1 (0.4–3.3); 0.866 1.2 (0.5–3.3); 0.665	19.67 0.0002 0.1610	36.3 (6.9–190.9); <0.001 0.4 (0.1–1.9); 0.271 1.1 (0.3–3.4); 0.898	29.62 <0.0001 0.2687
Both 3rd month and 4–5 month features correct—supine position Prematurity Sex	14.9 (4.2–52.2); <0.001 0.4 (0.1–1.7); 0.233 1.1 (0.4–3.3); 0.797	22.47 0.0001 0.1975	7.9 (2.5–24.8); <0.001 0.7 (0.2–2.1); 0.498 1.2 (0.5–3.0); 0.721	14.61 0.0022 0.1196	17.5 (4.4–68.5); <0.001 0.2 (0.4–1.0); 0.047 1.0 (0.3–3.0); 0.995	23.60 <0.0001 0.2141
Both 3rd month and 4–5 month features correct—prone position Prematurity Sex	14.8 (4.5–48.5); <0.001 0.6 (0.2–2.1); 0.390 1.1 (0.4–3.2); 0.845	24.41 <0.0001 0.2145	4.6 (1.6–12.8); 0.004 0.9 (0.3–2.5); 0.799 1.1 (0.4–2.8); 0.795	9.02 0.0290 0.0739	16.1 (4.6–56.1); <0.001 0.3 (0.1–1.2); 0.087 1.0 (0.3–2.9); 0.963	24.94 <0.0001 0.2263

**Table 3:** The influence of the partial motor elements (qualitative assessment) in the 3rd month of life, in the supine position, on achieving of the side sit and the oblique sit in the 7–8 months of life and final assessment at 12 months (walking). The results were adjusted for prematurity and sex; *n* = 93. The most significant results (with the highest OR values) are marked in gray.

Elements of Qualitative Assessment— Supine Position	Side Sit OR and 95%CI; *p* > [z]	LR chi2 (3) Prob > chi2 Pseudo R2	Oblique Sit OR and 95%CI; *p* > [z]	LR chi2 (3) Prob > chi2 Pseudo R2	Final Assessment at 12 Months OR and 95%CI; *p* > [z]	LR chi2 (3) Prob > chi2 Pseudo R2
Head symmetry Prematurity Sex	5.8 (2.0–17.4); 0.002 0.6 (0.2–2.1); 0.471 1.0 (0.4–2.5); 0.931	10.63 0.0139 0.0935	4.8 (1.6–14.0); 0.004 0.8 (0.3–2.3); 0.693 1.0 (0.4–2.6); 0.934	8.80 0.0321 0.0721	3.2 (1.1–9.3); 0.038 0.4 (0.1–1.5); 0.204 0.9 (0.3–2.3); 0.792	5.20 0.1580 0.0471
Shoulder in a balance between external and internal rotation—R Prematurity Sex	8.4 (3.0–23.7); <0.001 0.6 (0.2–2.1); 0.458 1.2 (0.4–3.2); 0.770	17.94 0.0005 0.1577	5.8 (2.2–15.6); <0.001 0.8 (0.3–2.4); 0.706 1.2 (0.5–3.1); 0.683	13.46 0.0037 0.1102	5.2 (1.9–14.4); 0.002 0.4 (0.1–1.4); 0.165 1.0 (0.4–2.7); 0.988	11.40 0.0098 0.1034
Shoulder in a balance between external and internal rotation—L Prematurity Sex	13.2 (3.6–47.9); <0.001 0.5 (0.1–1.7); 0.250 0.9 (0.3–2.6); 0.888	18.91 0.0003 0.1662	5.2 (1.6–16.2); 0.005 0.8 (0.2–2.2); 0.607 1.0 (0.4–2.6); 0.943	8.74 0.0329 0.0716	14.6 (3.7–58.3); <0.001 0.2 (0.1–1.0); 0.056 0.8 (0.3–2.3); 0.698	19.56 0.0002 0.1775
Wrist in an intermediate position—R Prematurity Sex	8.9 (2.7–28.9); <0.001 0.5 (0.1–1.7); 0.270 0.9 (0.3–2.5); 0.859	15.05 0.0018 0.1323	5.03 (1.7–15.1); 0.004 0.7 (0.2–2.1); 0.552 1.0 (0.4–2.5); 0.970	9.01 0.0292 0.0737	9.9 (2.8–35.0); <0.001 0.2 (0.1–1.1); 0.062 0.8 (0.3–2.2); 0.661	16.06 0.0011 0.1457
Wrist in an intermediate position—L Prematurity Sex	25.3 (2.8–231.3); 0.004 0.6 (0.2–2.1); 0.430 1.0 (0.4–2.8); 0.946	13.43 0.0038 0.1180	16.2 (1.8–142.4); 0.012 0.8 (0.3–2.4); 0.691 1.1 (0.4–2.8); 0.833	10.08 0.0179 0.0826	44.2 (4.0–492.3); 0.002 0.3 (0.1–1.3); 0.096 0.9 (0.3–2.6); 0.883	17.41 0.0006 0.1580
Palm in an intermediate position—R Prematurity Sex	7.2 (1.3–40.4); 0.025 0.8 (0.3–2.4); 0.688 1.0 (0.4–2.5); 0.994	5.70 0.1270 0.0501	2.5 (0.5–12.0); 0.255 1.0 (0.4–2.7); 0.933 1.1 (0.4–2.5); 0.886	1.35 0.7184 0.0110	9.5 (1.6–56.7); 0.014 0.4 (0.1–1.6); 0.210 0.9 (0.3–2.4); 0.832	7.96 0.0469 0.0722
Palm in an intermediate position—L Prematurity Sex	25.3 (2.8–231.3); 0.004 0.6 (0.2–2.1); 0.430 1.0 (0.4–2.8); 0.946	13.43 0.0038 0.1180	16.2 (1.8–142.4); 0.012 0.8 (0.3–2.4); 0.691 1.1 (0.4–2.8); 0.833	10.08 0.0179 0.0826	44.2 (4.0–492.3); 0.002 0.3 (0.1–1.3); 0.096 0.9 (0.3–2.6); 0.883	17.41 0.0006 0.1580
Thumb outside—R Prematurity Sex	7.2 (1.3–40.4); 0.025 0.8 (0.3–2.4); 0.688 1.0 (0.4–2.5); 0.994	5.70 0.1270 0.0501	2.5 (0.5–12.0); 0.255 1.0 (0.4–2.7); 0.933 1.1 (0.4–2.5); 0.886	1.35 0.7184 0.0110	9.5 (1.6–56.7); 0.014 0.4 (0.1–1.6); 0.210 0.9 (0.3–2.4); 0.832	7.96 0.0469 0.0722
Thumb outside—L Prematurity Sex	25.3 (2.8–231.3); 0.004 0.6 (0.2–2.1); 0.430 1.0 (0.4–2.8); 0.946	13.43 0.0038 0.1180	16.2 (1.8–142.4); 0.012 0.8 (0.3–2.4); 0.691 1.1 (0.4–2.8); 0.833	10.08 0.0179 0.0826	44.2 (4.0–492.3); 0.002 0.3 (0.1–1.3); 0.096 0.9 (0.3–2.6); 0.883	17.41 0.0006 0.1580
Spine in segmental extension Prematurity Sex	7.2 (1.3–40.4); 0.025 0.8 (0.3–2.4); 0.688 1.0 (0.4–2.5); 0.994	5.70 0.1270 0.0501	2.5 (0.5–12.0); 0.255 1.0 (0.4–2.7); 0.933 1.1 (0.4–2.5); 0.886	1.35 0.7184 0.0110	9.5 (1.6–56.7); 0.014 0.4 (0.1–1.6); 0.210 0.9 (0.3–2.4); 0.832	7.96 0.0469 0.0722
Pelvis extended Prematurity Sex	18.0 (4.6–70.4); <0.001 0.3 (0.1–1.3); 0.117 0.9 (0.3–2.7); 0.913	23.56 <0.0001 0.2070	4.4 (1.5–13.0); 0.007 0.7 (0.2–2.1); 0.521 1.0 (0.4–2.6); 0.923	7.76 0.0512 0.0636	14.2 (3.6–56.0); <0.001 0.2 (0.0–0.9); 0.035 0.8 (0.3–2.4); 0.750	19.87 0.0002 0.1803
Lower limb situated in moderate external rotation and lower limb bent at the right angle at hip and knee joints—R Prematurity Sex	13.2 (2.3–75.9); 0.004 0.6 (0.2–1.9); 0.355 0.9 (0.4–2.5); 0.901	10.70 0.0135 0.0940	8.2 (1.5–44.4); 0.015 0.8 (0.2.–2.0); 0.614 1.0 (0.4–2.5); 0.959	7.38 0.0608 0.0604	24.1 (3.3–175.6); 0.002 0.2 (0.0–1.2); 0.075 0.8 (0.3–2.3); 0.726	14.96 0.0018 0.1358
Lower limb situated in moderate external rotation and lower limb bent at the right angle at hip and knee joints—L Prematurity Sex	13.5 (2.6–70.7); 0.002 0.7 (02–2.3); 0.586 1.0 (0.4–2.8); 0.932	12.46 0.0060 0.1095	4.9 (1.2–20.8); 0.030 0.9 (0.3–2.6); 0.910 1.1 (0.4–2.7); 0.840	5.26 0.1540 0.0430	19.1 (3.3–110.7); 0.001 0.4 (0.1–1.4); 0.151 0.9 (0.3–2.6); 0.902	15.80 0.0012 0.1434
Foot in an intermediate position—R Prematurity Sex	8.1 (1.9–34.2); 0.005 0.5 (0.1–1.8); 0.300 1.0 (0.4–2.5); 0.935	9.15 0.0274 0.0804	4.8 (1.2–18.9); 0.025 0.7 (0.2–2.2); 0.573 1.0 (0.4–2.5); 0.927	5.54 0.1362 0.0454	15.3 (2.8–85.1); 0.002 0.2 90.0–1.0); 0.057 0.8 (0.3–2.3); 0.751	13.91 0.0030 0.1262
Foot in an intermediate position—L Prematurity Sex	9.1 (1.9–44.0); 0.006 0.6 (0.2–1.9); 0.352 1.1 (0.4–2.9); 0.843	8.75 0.0329 0.0769	3.2 (0.8–13.2); 0.106 0.8 (0.3–2.4); 0.766 1.1 (0.5–2.7); 0.800	2.73 0.4351 0.0224	16.7 (2.7–103.9); 0.003 0.2 (0.0–1.1); 0.074 1.0 (0.4–2.7); 0.991	12.99 0.0047 0.1178

**Table 4:** The influence of the partial motor elements (qualitative assessment) in the 3rd month of life, in the prone position, on achieving of the side sit and the oblique sit at 7–8 months and the final assessment at 12 months (walking). The results were adjusted for prematurity and sex; *n* = 93. The most significant results (with the highest OR values) are marked in gray.

Elements of Qualitative Assessment— Prone Position	Side Sit OR and 95%CI; *p* > [z]	LR chi2 (3) Prob > chi2 Pseudo R2	Oblique Sit OR and 95%CI; *p* > [z]	LR chi2 (3) Prob > chi2 Pseudo R2	Final Assessment at 12 Months OR and 95%CI; *p* > [z]	LR chi2 (3) Prob > chi2 Pseudo R2
Isolated head rotation Prematurity Sex	5.8 (2.1–16.2); 0.001 0.6 (0.2–1.9); 0.378 1.0 (0.4–2.8); 0.938	12.09 0.0071 0.1063	7.0 (2.5–19.8); <0.001 0.6 (0.2–2.0); 0.461 1.1 (0.4–3.0); 0.789	15.38 0.0015 0.1260	4.8 (1.7–13.7); 0.003 0.4 (0.1–1.3); 0.121 0.9 (0.3–2.5); 0.858	10.03 0.0183 0.0910
Arm in front—R Prematurity Sex	8.4 (2.7–25.6); <0.001 0.6 (0.2–2.0); 0.380 1.2 (0.4–3.2); 0.743	15.51 0.0014 0.1363	3.6 (1.3–10.1); 0.013 0.8 (0.3–2.4); 0.750 1.2 (0.5–2.9); 0.728	6.40 0.0937 0.0524	8.9 (2.8–28.8); <0.001 0.3 (0.1–1.2); 0.094 1.0 (0.4–3.0); 0.921	16.22 0.0010 0.1472
Arm in front—L Prematurity Sex	5.2 (1.8–14.6); 0.002 0.7 (0.2–2.1); 0.484 1.0 (0.4–2.6); 0.981	10.08 0.0179 0.0886	3.1 (1.2–8.4); 0.024 0.9 (0.3–2.4); 0.791 1.1 (0.4–2.6); 0.888	5.2 0.1565 0.0427	4.0 (1.4–11.5); 0.010 0.4 (0.1–1.4); 0.169 0.9 (0.3–2.3); 0.808	7.71 0.0525 0.0699
Palm loosely open—R Prematurity Sex	23.5 (2.6–207.5); 0.005 0.8 (0.2–2.6); 0.722 1.3 (0.5–3.4); 0.640	13.15 0.0043 0.1156	16.11 (1.8–140.2); 0.012 1.0 (0.3–2.8); 0.969 1.3 (0.5–3.2); 0.589	10.15 0.0174 0.0831	31.5 (3.3–297.4); 0.003 0.4 (0.1–1.6); 0.210 1.2 (0.4–3.2); 0.772	15.93 0.0012 0.1445
Palm loosely open—L Prematurity Sex	7.0 (1.2–39.3); 0.026 0.9 (03–2.8); 0.911 1.1 (0.4–2.9); 0.797	5.63 0.1312 0.0495	2.5 (0.5–12.2); 0.244 1.1 (0.0.4–2.9); 0.886 1.1 (0.5–2.7); 0.790	1.40 0.7047 0.0115	8.2 (1.4–46.8); 0.018 0.6 (0.2–1.8); 0.342 1.0 (0.4–2.7); 0.943	7.26 0.0640 0.0659
Thumb outside—R Prematurity Sex	25.3 (2.8–231.3); 0.004 0.6 (0.2–2.1); 0.430 1.0 (0.4–2.8); 0.946	13.43 0.0038 0.1180	16.2 (1.8–142.4); 0.012 0.8 (0.3–2.4); 0.691 1.1 (0.4–2.8); 0.833	10.08 0.0179 0.0826	44.2 (4.0–492.3); 0.002 0.3 (0.1–1.3); 0.096 0.9 (0.3–2.6); 0.883	17.41 0.0006 0.1580
Thumb outside—L Prematurity Sex	7.2 (1.3–40.4); 0.025 0.8 (0.3–2.4); 0.688 1.0 (0.4–2.5); 0.994	5.70 0.1270 0.0501	2.5 (0.5–12.2); 0.244 1.1 (0.0.4–2.9); 0.886 1.1 (0.5–2.7); 0.790	1.35 0.7184 0.0110	9.5 (1.6–56.7); 0.014 0.5 (0.1–1.6); 0.210 0.9 (0.3–2.4); 0.832	7.96 0.0469 0.0722
Spine in segmental extension Prematurity Sex	5.9 (2.2–16.1); 0.001 0.6 (0.2–2.0); 0.427 1.0 (0.4–2.8); 0.909	12.86 0.0050 0.1130	5.2 (2.0–13.9); 0.001 0.8 (0.2–2.2); 0.610 1.1 (0.4–2.9); 0.791	12.05 0.0072 0.0987	3.8 (1.4–10.3); 0.009 0.4 (0.1–1.4); 0.166 0.9 (0.4–2.5); 0.900	7.82 0.0498 0.0710
Scapula situated in the medial position—R Prematurity Sex	5.1 (1.7–15.4); 0.004 0.7 (0.2–2.1); 0.506 1.1 (0.4–2.9); 0.847	8.72 0.0332 0.0767	3.2 (1.1–9.2); 0.033 0.8 (0.3–2.5); 0.802 1.1 (0.5–2.8); 0.776	4.71 0.1941 0.0386	5.1 (1.6–15.8); 0.005 0.4 (0.1–1.4); 0.151 1.0 (0.4–2.6); 0.983	9.00 0.0293 0.0816
Scapula situated in the medial position—L Prematurity Sex	3.3 (1.2–9.1); 0.021 0.8 (0.3–2.40; 0.676 1.0 (0.4–2.6); 0.935	5.34 0.1488 0.0469	2.7 (1.0–7.3); 0.048 0.9 (0.3–2.6); 0.907 1.1 (0.4–2.7); 0.827	3.98 0.2633 0.0326	2.4 (0.9–6.8); 0.094 0.5 (0.2–1.6); 0.261 0.9 (0.4–2.4); 0.884	3.64 0.3029 0.0330
Pelvis in an intermediate position Prematurity Sex	13.3 (3.8–46.2); <0.001 0.4 (0.1–1.5); 0.160 0.9 (0.3–2.5); 0.809	20.86 0.0001 0.1833	5.0 (1.7–14.5); 0.003 0.7 (02–2.1); 0.498 1.0 (0.4–2.50); 0.993	9.35 0.0250 0.0766	10.7 (3.1–37.6); <0.001 0.2 (0.1–1.0); 0.047 0.8 (0.3–2.2); 0.662	17.54 0.0005 0.1592
Lower limbs situated loosely—R Prematurity Sex	14.7 (2.9–73.8); 0.001 0.4 (0.1–1.6); 0.204 1.0 (0.4–2.5); 0.920	13.91 0.0030 0.1222	8.1 (1.8–36.1); 0.006 0.6 (0.2–2.0); 0.437 1.0 (0.4–2.6); 0.938	9.19 0.0268 0.0753	40.6 (4.3–381.4); 0.001 0.1 (0.0–1.0); 0.044 0.8 (0.3–2.3); 0.729	20.25 0.0002 0.1838
Lower limbs situated loosely—L Prematurity Sex	8.3 (2.1–31.8); 0.002 0.6 (0.2–2.1); 0.447 1.0 (0.4–2.8); 0.929	10.70 0.0135 0.0940	3.4 (1.0–11.9); 0.050 0.9 (0.3–2.5); 0.816 1.1 (0.4–2.6); 0.839	4.02 0.2589 0.0330	12.6 (2.9–55.6); 0.001 0.3 (0.1–1.3); 0.098 0.9 (0.3–2.6); 0.893	14.70 0.0021 0.1334
Foot in an intermediate position—R Prematurity Sex	7.9 (1.3–46.7); 0.023 0.7 (0.2–2.1); 0.512 1.0 (0.4–2.5); 0.954	5.98 0.1124 0.0526	5.2 (0.9–29.6); 0.064 0.9 (0.3–2.4); 0.778 1.0 (0.4–2.5); 0.919	3.92 0.2707 0.0321	12.2 (1.8–82.2); 0.010 0.4 (0.1–1.4); 0.129 0.9 (0.3–2.3); 0.794	8.91 0.0306 0.0808
Foot in an intermediate position—L Prematurity Sex	7.3 (1.3–41.2); 0.025 0.8 (0.3–2.4); 0.718 1.1 (0.4–2.8); 0.832	5.75 0.1245 0.0505	2.5 (0.5–12.3); 0.248 1.0 (0.4–2.7); 0.978 1.1 (0.5–2.7); 0.808	1.38 0.7093 0.0113	9.5 (1.6–56.9); 0.014 0.5 (0.1–1.6); 0.225 1.0 (0.4–2.7); 0.970	7.96 0.0469 0.0722

## Data Availability

The datasets used and/or analyzed during the current study are available from the corresponding author on reasonable request.
